# Parent–Youth Attachment Insecurity and Informant Discrepancies of Intrafamilial Aggression

**DOI:** 10.1007/s10578-023-01662-2

**Published:** 2024-02-15

**Authors:** Emily M. Thornton, Sebastian P. Dys, Carlos Sierra Hernandez, Ryan J. Smith, Marlene M. Moretti

**Affiliations:** 1 Department of Psychology, University of Guelph, 50 Stone Rd E, Guelph, ON N1G 2W1 Canada; 2 Department of Psychology, Simon Fraser University, 8888 University Drive, Burnaby, BC V5A 1S6 Canada

**Keywords:** Attachment, Aggression, Parent, Adolescent, Informant discrepancies

## Abstract

This study investigated how youth attachment anxiety and avoidance are associated with informant discrepancies of intrafamilial aggression within families where youth have clinically significant mental health challenges (*N* = 510 youth–parent dyads). Using polynomial regressions, we tested whether youth attachment avoidance and anxiety moderated the absolute magnitude of the association between youth- and parent-reports of aggression toward each other. Furthermore, difference scores were computed to test whether youth attachment was associated with the direction of youths’ reports of the frequency of aggression relative to parents (i.e., did youth under- or over-report). Dyads’ reports of youth-to-parent aggression were more strongly related at high than low levels of attachment anxiety. Results also revealed that youth attachment anxiety was associated with youth over-reporting of youth-to-parent and parent-to-youth aggression (relative to parents), whereas attachment avoidance was associated with youth over-reporting parent-to-youth aggression (relative to parents). These findings highlight the importance of understanding the source of informant discrepancies in social-emotional development and family functioning.

## Introduction

The nature of people’s attachments with their primary caregivers is widely believed to shape their perceptions, interpretations, and responses to interpersonal behavior [1]. Different attachment styles may generate specific types of biases that influence social interactions. For example, individuals high in attachment anxiety appear to be better able to retrieve negative memories [2–4]. These attachment-related biases tend to be most pronounced during stressful conflicts with attachment figures [5]. Conflict between parents and youth are one such type of interaction that family members may perceive in diverging manners, depending on their attachment styles [6, 7]. Furthermore, unlike other outcomes (e.g., youth depression) for which discrepancies can be subject to contextual differences stemming from observing someone in different environments (e.g., at home versus at school), overt aggression between family members is not subject to these contextual effects as it is presumed to be equally visible to both informants. Yet, relatively little attention has been paid to the meaning of discrepancies between parents and youth in this domain.

The present study sought to address the gap in this literature by examining how youth attachment anxiety and avoidance related to the discrepancies between youth and parent reports of youth aggression toward parents as well as parent aggression toward youth. We examined these discrepancies from two perspectives: first, in terms of their magnitude—i.e., how strongly do parents and youth (dis)agree—and second, in terms directional differences—i.e., under what conditions do adolescents under- or over-report aggression, relative to their parents. Understanding the magnitude and direction of systematic discrepancies between youth and parent reports may hold important implications for researchers—who, for instance, may seek to understand diverging findings across informants—as well as clinicians—who, for example, may be interested in addressing discrepant perspectives about intrafamilial violence as a starting point for therapy.

### Attachment Anxiety and Avoidance

Youth high in attachment insecurity (e.g., attachment anxiety or avoidance) are unlikely to expect parents to respond with sensitivity to their bids for a safe haven and secure base when called upon during periods of stress and challenge [8]. Attachment anxiety involves the overactivation of the attachment system and is associated with worrying that others will not be responsive in times of needs and thus the escalated expression of one’s attachment needs [9, 10]. Conversely, attachment avoidance involves a deactivation of the attachment system and is associated with masking or muting the expression of one’s attachment needs [10]. As these forms of attachment insecurity are associated with the distorted expression of attachment needs, they may “miscue” others who in turn respond in ways that perpetuate the cycle of insecurity, locking family members into non-productive and conflictual interaction patterns. In contrast, attachment security is associated with the clear communication of attachment needs and thus promotes a shared understanding of interpersonal intentions and interactions, increasing the likelihood of sensitive responsiveness [11, 12].

### Attachment Insecurity and Perceptions of Interpersonal Conflict and Aggression

Research examining attachment-related biases among youth in their reporting of their own and others’ aggressive behavior is limited. Some studies have found that youth attachment security is related to more agreement between parent and youth reports of conflict and externalizing problems [13–15], but few have examined the unique ways that attachment anxiety and avoidance influence such biases. Among the few, Berger et al. [13] found that attachment anxiety was associated with greater absolute discrepancies (i.e., disagreements in either direction) between youth and parent reports of youth externalizing problems. They also found attachment anxiety was related to youths reporting higher levels of externalizing problems relative to their parents’ reports. Conversely, adolescent attachment avoidance was related to lower levels of absolute agreement between youth and mother (but not father) reports, and no directional differences in reporting higher or lower levels of symptoms than their parents.

Among adults, attachment insecurity has been associated with more disagreement between romantic partners’ reports of conflict in their relationship. For example, Campbell et al. [16] found that anxiously attached partners overreported the frequency and severity of relationship conflict, compared with their romantic partner. In another study, Overall et al. [17] found that participants’ attachment avoidance was associated with overreporting how intense their partners’ negative emotions were during conflict discussions. Similarly, Ehrlich et al. [18] found that attachment security was associated with smaller discrepancies in couples’ reports of marital conflict. In sum, extant studies appear to indicate that attachment anxiety and avoidance are related to greater discrepancies between informants. Furthermore, these findings suggest that attachment anxiety is generally associated with over-reporting one’s own externalizing and conflict behavior, whereas attachment avoidance is linked to over-reporting others’ negative emotional responding, which may generalize to aggressive behaviors.

### Statistical Approaches to Assessing Informant Discrepancies

In recent years, investigations on informant discrepancies have garnered increased attention and employed more advanced analytic approaches such as polynomial regression (see [19]). Polynomial regression allows researchers to test whether scores from one reporter increase or decrease in a stepwise manner with scores from a second reporter. Unlike most other tests of informant discrepancies, it allows for testing whether this relation follows a linear or higher-order function (e.g., quadratic, cubic, [20]). Polynomial regression analyses have increasingly been used in clinical psychology to understand the factors that are associated with discrepancies between parent–youth reports, for example by examining how disorganized attachment is associated with discrepancies between youth and parent reports of youths’ internalizing and externalizing symptoms [14]. Here, we employed polynomial regression analyses to investigate whether youth attachment anxiety and avoidance moderate the *magnitude of the association* between youth and parent reports of aggressive behaviors.

In addition to our polynomial regression analyses, we also examined whether youth anxiety and avoidance were related to youth under- or over-reporting instances of aggression, compared with parent reports, via difference scores. These have long been used to assess directional differences between parent and children reports of various constructs (e.g., [13]). Difference scores add important information beyond polynomial regression analyses because, even though a strong association may exist between reporters (indicating their scores increase or decrease in relation to each other), difference scores can show whether one reporter consistently provides higher or lower estimates than the other. Although most past research has used either polynomial regression or analysis of difference scores, applying both alongside each other provides a unique opportunity to examine both the (1) the *magnitude of discrepancies* between different reporters on the same construct, and (2) *directional* differences between reporters, helping to explain why one reporter may systematically provide higher or lower estimates relative to the other. Understanding both types of systematic differences may be valuable for researchers and clinicians when trying to make decisions around which informant type to collect data from, or to make sense of discrepancies in mean levels or covariances between youth and parent reports.

#### The Present Study

The aim of the present study was to examine whether youth attachment anxiety and avoidance were associated with discrepancies between youth and parent reports of youth-to-parent and parent-to-youth aggression. Using a sample of youth with clinically significant mental health problems and their parents, we examined the *magnitude* and *direction* of these discrepancies via two approaches. First, we used polynomial regression analyses to investigate whether youth attachment anxiety and avoidance moderated the magnitude of the relation between youth and parent reports of intrafamilial aggression. Second, in analyses using difference scores, we tested whether youth attachment anxiety and attachment avoidance were associated with the direction of their reports on intrafamilial aggression (i.e., did youth under- or over-report) relative to their parents. Unlike previous studies, informants provided ratings on behaviors that only occur between the informants (i.e., aggression between youth and their parents), thus informant discrepancies could not be attributed to domain differences (e.g., observing youth at home versus school). In terms of absolute discrepancies, we expected that youth attachment anxiety and avoidance would be related to greater discrepancies between youth and parent reports of aggression in their relationship. In terms of directional differences, based on prior research, we expected attachment anxiety to be related to youth over-reporting of youth-to-parent aggression (relative to their parents’ reports), whereas we expected attachment avoidance to be related to youth over-reporting of parent-to-youth aggression (relative to their parents’ reports). We did not have specific hypotheses about how high attachment anxiety would relate to directional differences in youth reports of parent-to-youth aggression, or how attachment avoidance would relate to directional differences in youth reports of youth-to-parent aggression, as the findings on these relations have been less clear.

## Method

### Participants and Procedure

We obtained ethics approval for this study from the Research Ethics Board at Simon Fraser University, protocol #2011s0284. Participants were 510 birth parents (*M*_age_ = 42.64 years, *SD* = 6.86, range = 27.00–65.00; 86% mothers) and their children (*M*_age_ = 13.96 years, *SD* = 2.36, range = 7.34–19.04; 56.1% female) referred by community mental health centers, schools, and hospitals to participate in a parenting intervention for caregivers of youth with severe emotional and behavioral difficulties (i.e., the Connect Parenting Program). Parents reported on their ethnicity (74.7% White; 11.4% Indigenous; 7.1% Asian; 3.9% “other ethnicity”) as did their youth (65.1% White; 15.2% Indigenous; 5.7% Asian; 8.6% “other” ethnicities). Data for this study consisted of baseline assessments prior to treatment. Exclusion criteria for this study included low cognitive functioning, severe psychopathology (e.g., psychosis), and low fluency in English. In cases where both parents of youth participated in the research (*N* = 7), maternal reports were retained to avoid dependencies in the data. Consent to participate was provided by parents and assent by youth.

### Measures

*Intrafamilial Aggression*. Participants completed the Revised Conflict Tactics Scale (CTS-2; [21]), a widely used questionnaire that assesses engagement in physical and psychological aggression. Past research has found support for the psychometric properties of the CTS-2, including good internal consistency, reliability, validity, and factor structure [21–24]. Due to questionnaire space limitations, we employed a modified version of the CTS-2 to capture perpetration and victimization within the family in the past 6 months. Specifically, parents and their teens reported perpetration and victimization through physical (7 items) and psychological aggression (9 items) using a 4-point scale (1 = *Never* to 4 = *Always*). Total scores for perpetration and victimization were computed for parents and youth by summing their responses on items tapping physical and psychological aggression. This procedure yielded scores tapping (1) youth-to-parent aggression reported by youth, (2) youth-to-parent aggression reported by parents, (3) parent-to-youth aggression reported by youth, and (4) parent-to-youth aggression reported by parents. These measures showed good internal reliability in our data for youth reports of their physical (α = 0.84) and psychological (α = 0.86) aggression, as well as their parents’ physical (α = 0.88) and psychological (α = 0.88) aggression. Similarly, good internal reliability was demonstrated in measures of parent reports of their physical (α = 0.73) and psychological (α = 0.82) aggression, as well as youth physical (α = 0.89) and psychological (α = 0.89) aggression. Previous studies using this modified version of the CTS-2 have found similarly strong reliability and validity indices [25, 26].

*Attachment Anxiety and Avoidance*. Youth completed the Adolescent Attachment Anxiety & Avoidance Inventory (AAAAI; [27]; previously the Comprehensive Adolescent Parent Attachment Inventory), a measure adapted from Brennan et al.’s [28] Experiences in Close Relationships Scale (ECRS). Consistent with other self-report measures of attachment, two super-ordinate factors consistently emerge from this 36-item measure: attachment anxiety and attachment avoidance [27, 29–32]. In this study, due to questionnaire space limitations, we employed a shortened version of the AAAAI, which included 16 items with the highest factor loadings from the original scale such as “I need a lot of reassurance that I am loved by my parent” (attachment anxiety) and “I try to avoid getting too close to my parent” (attachment avoidance). Items were rated using a 7-point scale (1 = *Strongly Disagree* to 7 = *Strongly Agree*). The scale has shown strong psychometric properties—including clear factor structure and strong convergent validity—in community and clinical samples [30, 33, 34]. Others who have used similar adaptations of the ECRS have also found strong psychometrics [35, 36]. In the present study, internal reliability for attachment anxiety and avoidance measures was strong (αs = 0.83 and 0.90, respectively).

### Data Analytic Plan

#### Polynomial Regression Analyses

Four hierarchical polynomial regression analyses were conducted, each predicting youth-reports of youth or parent aggression from parent-reports of the same aggressive behavior. For each model, parent and youth sex and age were entered into the first step of each regression to control for potential confounds. For each model, the second step included five terms: parent reported aggression, youth attachment anxiety or avoidance, the two aforementioned terms squared, and the interaction term between parent-reported aggression. In two of the models, youth attachment anxiety was included as a predictor in this step and in two other models, youth attachment avoidance was included. The third and final step in the hierarchical regressions included higher order terms. We included the squared terms (in Step 2) to prevent interaction terms from reflecting quadratic effects of informant reports, which is important to consider when one cannot assume informant agreement is strictly linear (see [37]). Furthermore, in line with recommendations from Edwards [20], we modeled interactions with these quadratic terms (in Step 3), to avoid underestimating the complexity of associations within our data. Significant and marginally significant interaction terms were interpreted using the Johnson–Neyman (JN) technique [38], which allowed us to identify the values of the moderator (i.e., regions of significance) at which the relation between the predictor and dependent variable is significant. Polynomial regression analyses had sufficient power (0.80) to detect relatively small effects *f*^2^ = 0.03, using a two-tailed test with a *p* value of 0.05—for comparison, Cohen [39] labeled *f*^2^ = 0.02 as a small effect and *f*^2^ = 0.15 as a medium effect.

*Difference Score Analyses* Multiple regression analyses were conducted using standardized youth-parent conflict discrepancy scores to examine whether youth attachment anxiety or avoidance was related to aggression under- or over-reporting relative to parents. Like our polynomial analyses, we ran four models, each predicting difference scores for each type of aggression (i.e., youth-to-parent and parent-to-youth) from each type of attachment insecurity (i.e., anxiety and avoidance). In line with guidelines from De Los Reyes and Kazdin [40], difference scores were computed by standardizing all scores and subtracting youth reports from parent reports of aggression. We entered parent and youth sex and age as predictors in Step 1 to control for their potential effects. We entered either attachment anxiety or avoidance in Step 2. Analyses were conducted using IBM SPSS Statistics 25. The data that support all the findings of this study are available from the corresponding author upon reasonable request.

## Results

### Descriptive Statistics

Descriptive statistics and bivariate correlations are summarized in Table 1 and show that parents and youth reported slightly higher levels of aggression perpetrated towards them compared to aggression perpetrated by them. Specifically, parent reports of youth-to-parent aggression (*M* = 3.22, *SD* = 0.98) exceeded youth reports of their own aggression (*M* = 2.93, *SD* = 0.82; *t*(450) = 6.57, *p* < 0.001). Similarly, youth reports of parent-to-youth aggression (*M* = 2.98, *SD* = 0.97) exceeded parent reports of their own aggression (*M* = 2.69, *SD* = 0.55, *t*(453) = 6.72, *p* < 0.001). Nonetheless, parent and youth reports of youth-to-parent and parent-to-youth aggression were significantly correlated (*r* = 0.47, *p* < 0.001 and *r* = 0.37, *p* < 0.001, respectively). Next, we describe the results from our polynomial regression analyses, starting with how attachment anxiety predicts informant discrepancies of youth-to-parent and then parent-to-youth aggression. We then report the next set of analyses, but with youth attachment avoidance. Thereafter, we report the results of parallel analyses using difference scores, presented in the same order as they were for polynomial regression analyses.

**Table 1:** Correlations and descriptive statistics of key study variables

	1	2	3	4	5	6	*Mean* (*SD*)	Observed range
Parent reports								
1. Youth aggression (toward parent)	–						3.22 (0.98)	2.00–8.00
2. Parent aggression (toward youth)	0.51***	–					2.70 (0.55)	2.00–5.97
Youth reports								
3. Youth aggression (toward parent)	0.47***	0.32***	–				2.93 (0.82)	2.00–7.21
4. Parent aggression (toward youth)	0.35***	0.37***	0.67***	–			2.98 (0.97)	2.00–8.00
5. Youth attachment anxiety	0.14**	0.14**	0.30***	0.28***	–		2.70 (1.28)	1.00–7.00
6. Youth attachment avoidance	0.14**	0.05	0.19***	0.35***	0.22***	–	3.72 (1.49)	1.00–6.89

**p < 0.01; ***p < 0.001

### Polynomial Regression Analyses

#### Attachment Anxiety and Youth and Parent Reports of Aggressive Behavior

**Fig. 1:**
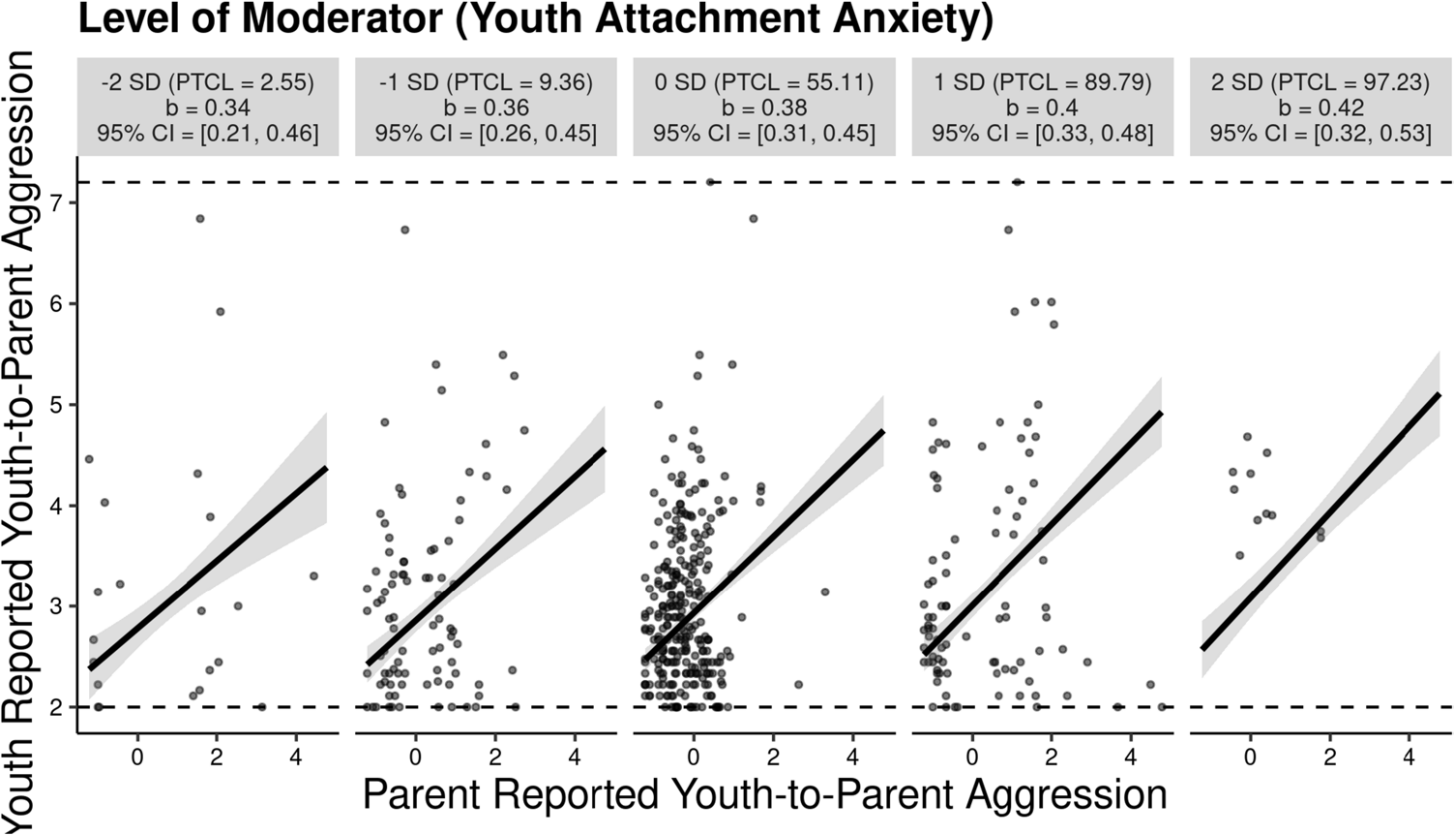
Youth reports of youth aggression as a function of parent reports of youth aggression and youth attachment anxiety

*Youth-to-Parent Aggression* Results from the hierarchical polynomial regression analyses (see Table 2) showed that parent reports of youth-to-parent aggression significantly predicted youth reports of their own aggressive behavior, as did the quadratic term of parent reports of youth aggression. Youth attachment anxiety, but not its quadratic term, significantly predicted youth reports of youth-to-parent aggression. Key to our predictions, the interaction term between parent report of youth aggression and attachment anxiety was also significant (*b* = 0.08, *SE* = 0.03, *p* = 0.007), but not in our expected direction—the association between parent and youth reports of youth aggression was stronger at higher rather than lower levels of youth attachment anxiety. We illustrated this effect using the *interActive* plotting utility ([41], see Fig. 1). The Johnson–Neyman technique indicated that the effect of parent reports of youth aggression on youth reports of youth aggression was significant across all values of youth attachment anxiety.

**Table 2:** Polynomial regression predicting youth reports of intrafamilial aggression from parent reports of intrafamilial aggression and youth attachment anxiety

Parameter	Youth-to-parent aggression				Parent-to-youth aggression			
	*b*	*SE*	*p*	Δ*R*^2^	*b*	*SE*	*p*	Δ*R*^2^
Step 1				0.004				0.019
Parent sex	− 0.018	0.114	0.878		− 0.196	0.132	0.138	
Youth sex	0.097	0.079	0.222		0.132	0.092	0.154	
Parent age	− 0.001	0.006	0.890		− 0.001	0.007	0.892	
Youth age	0.000	0.019	0.990		0.040	0.022	0.065	
Step 2				0.287***				0.196***
Parent report of aggression	0.452	0.046	0.000		0.753	0.096	0.000	
Parent report of aggression squared	− 0.070	0.022	0.002		− 0.240	0.074	0.001	
Youth attachment anxiety	0.148	0.029	0.000		0.159	0.036	0.000	
Youth attachment anxiety squared	− 0.034	0.020	0.082		0.001	0.024	0.963	
Parent report of aggression × youth attachment anxiety	0.081	0.030	0.007		0.085	0.059	0.152	
Step 3				0.000				0.001
Parent report of aggression squared × youth attachment anxiety	− 0.007	0.022	0.751		0.043	0.070	0.540	
Parent report of aggression × youth attachment anxiety squared	− 0.003	0.024	0.895		− 0.012	0.050	0.811	

Sex: 0 = female, 1 = male

***p < 0.001

*Parent-to-Youth Aggression* Results from the hierarchical polynomial regression (see Table 2) showed that parents’ reports of their own aggression and its quadratic term significantly predicted youth reports of parent-to-youth aggression. Youth attachment anxiety, but not its quadratic term, also predicted youth reports of parent-to-youth aggression. The interaction term between parent reports of parent-to-youth aggression and youth attachment anxiety, however, was not significant, indicating that youth attachment anxiety was not related to the strength of agreement between informants on parent-to-youth aggression. Parent and youth age and sex were not significant predictors of youth reports of parent-to-youth aggression.

#### Attachment Avoidance and Youth and Parent Reports of Aggressive Behavior

*Youth-to-Parent Aggression* Parent reports of youth-to-parent aggression and its quadratic term significantly predicted youth reports of youth-to-parent aggression (see Table 3). Attachment avoidance also significantly predicted youth reports of youth-to-parent aggression. The squared term of attachment avoidance was not significant, however. The interaction between parent reports of youth aggression and youth attachment avoidance fell below significance (*b* = − 0.04, *SE* = 0.02, *p* = 0.08), but was in the hypothesized direction (i.e., we expected the association between parent and youth reports of youth aggression to be stronger at lower levels of youth attachment avoidance). Parent and youth age and sex were not significant predictors of youth reports of youth-to-parent aggression.

**Table 3:** Polynomial regression predicting youth reports of intrafamilial aggression from parent reports of intrafamilial aggression and youth attachment avoidance

Parameter	Youth-to-parent aggression				Parent-to-youth aggression			
	*b*	*SE*	*p*	Δ*R*^2^	*b*	*SE*	*p*	Δ*R*^2^
Step 1				0.004				0.017
Parent sex	− 0.020	0.114	0.860		− 0.175	0.132	0.186	
Youth sex	0.098	0.079	0.216		0.132	0.092	0.154	
Parent age	− 0.001	0.006	0.872		− 0.001	0.007	0.921	
Youth age	0.000	0.019	0.998		0.038	0.022	0.080	
Step 2				0.251***				0.257***
Parent report of aggression	0.443	0.046	0.000		0.778	0.092	0.000	
Parent report of aggression squared	− 0.048	0.022	0.031		− 0.216	0.071	0.002	
Youth attachment avoidance	0.073	0.024	0.003		0.199	0.028	0.000	
Youth attachment avoidance squared	− 0.004	0.014	0.805		0.039	0.017	0.024	
Parent report of aggression × youth attachment avoidance	− 0.042	0.024	0.083		− 0.088	0.055	0.110	
Step 3				0.001				0.000
Parent report of aggression squared × youth attachment avoidance	− 0.007	0.015	0.663		− 0.069	0.071	0.328	
Parent report of aggression × youth attachment avoidance squared	0.011	0.016	0.479		0.016	0.041	0.690	

Sex: 0 = female, 1 = male

***p < 0.001

*Parent-to-Youth Aggression* Parent reports of parent aggression and its quadratic term significantly predicted youth reports of parent aggression (see Table 3). Attachment avoidance was also a significant predictor. The quadratic term of attachment avoidance did not significantly predict youth reports of parent aggression, however. Additionally, the interaction between parent reports of parent aggression and youth attachment avoidance was not significant (*b* = − 0.09, *SE* = 0.06, *p* = 0.11), but was consistent with the direction we predicted (i.e., that youth attachment avoidance would be associated with more informant discrepancies). Youth and parent age and sex were not significant predictors of youth reports of parent-to-youth aggression.

### Difference Score Analyses

#### Attachment Anxiety and Youth and Parent Reports of Aggressive Behavior

*Youth-to-Parent Aggression* Consistent with prior findings, our results revealed that the effect youth attachment anxiety was significant, with youth reporting higher levels of youth-to-parent aggression relative to parents (*b* = − 0.100, *SE* = 0.039, *p* = 0.011; see Table 4). In terms of control variables, we found youth sex was significant, with girls tending to report higher levels of their aggression relative to their parents (*b* = − 0.216, *SE* = 0.100, *p* = 0.031).

**Table 4:** Regression analyses predicting standardized difference scores (parent minus youth report) in intrafamilial aggression from youth attachment anxiety

Parameter	Youth-to-parent aggression				Parent-to-youth aggression			
	*b*	*SE*	*p*	Δ*R*^2^	*b*	*SE*	*p*	Δ*R*^2^
Step 1				0.016				0.015
Parent sex	− 0.170	0.144	0.238		− 0.064	0.155	0.677	
Youth sex	− 0.216	0.100	0.031		− 0.220	0.108	0.043	
Parent age	− 0.004	0.008	0.592		− 0.009	0.009	0.292	
Youth age	0.007	0.024	0.781		− 0.004	0.026	0.882	
Step 2				0.015*				0.012*
Youth attachment anxiety	− 0.100	0.039	0.011		− 0.099	0.043	0.021	

Sex: 0 = female, 1 = male

*p < 0.05

*Parent-to-Youth Aggression* Findings revealed that the effect of youth attachment anxiety was significant, with youth reporting higher levels of parent-to-youth aggression relative to parents (*b* = − 0.099, *SE* = 0.043, *p* = 0.021; see Table 4). In terms of control variables, only the effect sex was significant, with girls tending to report higher level of parental aggression relative to parents (*b* = − 0.220, *SE* = 0.108, *p* = 0.043).

#### Attachment Avoidance and Youth and Parent Reports of Aggressive Behavior

*Youth-to-Parent Aggression* We found no effect of youth attachment avoidance on difference scores of youth-to-parent aggression (*p* = 0.318; see Table 5). Again, the effect of sex was significant, with girls reporting higher levels of their own aggression relative to their parents (*b* = − 0.220, *SE* = 0.100, *p* = 0.027).

**Table 5:** Regression analyses predicting standardized difference scores (parent minus youth report) in intrafamilial aggression from youth attachment avoidance

Parameter	Youth-to-parent aggression				Parent-to-youth aggression			
	*b*	*SE*	*p*	Δ*R*^2^	*b*	*SE*	*p*	Δ*R*^2^
Step 1				0.016				0.015
Parent sex	− 0.161	0.143	0.261		− 0.062	0.155	0.690	
Youth sex	− 0.220	0.100	0.027		− 0.224	0.108	0.039	
Parent age	− 0.004	0.008	0.635		− 0.009	0.009	0.314	
Youth age	0.006	0.023	0.803		− 0.004	0.026	0.881	
Step 2				0.002				0.062***
Youth attachment avoidance	− 0.034	0.034	0.318		− 0.198	0.037	0.000	

Sex: 0 = female, 1 = male

***p < 0.001

*Parent-to-Youth Aggression* In line with previous findings, we found that the effect of youth attachment avoidance was significant, with youth reporting more parent-to-youth aggression relative to parents (*b* = − 0.198, *SE* = 0.037, *p* < 0.001; see Table 5). In terms of control variables, we found an effect of gender, with girls tending to report higher levels of parent-to-youth aggression relative to their parents (*b* = − 0.224, *SE* = 0.108, *p* = 0.039).

## Discussion

A rapidly growing body of research has highlighted the role of systematic discrepancies between informants, especially parents and youth, when assessing youth social-emotional functioning [42, 43]. With a clinical sample of youth and their parents, the current study sought to examine how two aspects of youth attachment insecurity (anxiety and avoidance) were associated with discrepancies of familial aggression between youth and parents in two ways: first, in terms of the magnitude of their discrepancy, and second, in terms of the direction of their discrepancy (i.e., whether youth under- or over-report relative to parents). Unlike previous studies, parent and adolescent dyads reported on aggression they perpetrated towards each other, meaning that discrepancies between their reports could not be attributed to domain differences (e.g., parents being unable to observe youth aggression at school as well as at home). We hypothesized that higher levels of youth attachment anxiety and attachment avoidance would be related to less absolute agreement on ratings of intrafamilial aggression. We also expected that, relative to parents, youth attachment anxiety would be related to over-reporting their own (i.e., youth-to-parent) aggression, and youth attachment avoidance would be related to over-reporting parents’ (i.e., youth-to-parent) aggression. The findings partially confirmed our hypotheses.

First, in contrast to our hypothesis, we found that youth attachment anxiety was related to more agreement between youth and parent reports of youth aggression toward their parents. One possible explanation is that attachment anxiety, which is characterized by amplified expressions of needs to attachment figures [9], may increase the visible incidents of conflictual interactions between youth and their parents and thereby increase their agreement about conflict in the relationship, particularly when conflict is instigated by youth [44]. In families where youth are high in attachment anxiety, parents and youth may share a heightened cognitive understanding of youth aggressive behavior and attachment needs, but parents may lack the capacities to respond to their adolescents’ needs sensitively. This may explain a relatively high degree of agreement within these dyads, while accounting for the relatively high level of attachment anxiety experienced by youths. Furthermore, we did not find a parallel significant effect for parents’ aggression toward their youth, although the effect was in the same direction (i.e., higher levels of attachment anxiety related to more agreement on parent-to-youth aggression). Here, we examined youth reports of attachment anxiety to their parents in relation to informant discrepancies but did not examine parent reports of youth attachment. Examining whether parents’ reports of youth attachment predict further discrepancies between informants makes for a promising future research question.

Second, we found that the relations between attachment avoidance and discrepancies between youth and parents’ reports of either type of aggression (i.e., youth-to-parent and parent-to-youth) fell below significance. Still, the observed effects were in line with our expected direction which were drawn from prior theory and research showing that attachment avoidance is associated with suppression in the expression of attachment needs. Masked or muted communication of attachment needs could constrict communication and undermine a shared view between youths and parents about the level of conflict in the relationship [44]. One potential explanation for these null findings is that youths’ own attachment avoidance may be systematically related to how they self-report their avoidance—for instance, youth who experience high levels of attachment avoidance may downplay their degree of avoidance, thereby attenuating statistical relations between self-reports of attachment avoidance and informant discrepancies. But it is also conceivable that the effects of attachment avoidance on informant discrepancies are relatively small in magnitude.

Next, moving beyond absolute discrepancies, we found that youth attachment insecurity was also related to the direction of their reports’ discrepancies of intrafamilial aggression relative to their parents. As predicted, we found youth attachment anxiety was related to youth over-reporting their perpetration of aggression toward their parents, relative to their parents’ reports. For youth high in attachment anxiety, their preoccupation with securing their parents attention and love may lead them to ruminate about past aggression toward their parents, consolidating these memories, and potentially exacerbating their perceptions of their misdemeanors toward their parents, deepening their attachment anxiety [4]. In contrast, we found that youth attachment avoidance was related to youth over-reporting their parents’ perpetration of aggression toward them, relative to their parents’ reports. This is not surprising given that attachment avoidance is associated with expectations that others will reject or respond punitively to their expression of attachment needs, thus priming their vigilance and perception of threat in others’ behaviors. These adolescents’ sensitivity to detecting such aggression is likely key in perpetuating their masked or muted expression of their attachment needs. In turn, their parents may lack awareness of the impact of their aggressive behavior on their teens and, consequently, fail to alter their behavior [45].

The current study is unique in multiple ways. First, it involved assessing informant discrepancies of aggression between youth and parents—behaviors both parties would be directly involved in—which allows us to rule out domain differences as a source of informant discrepancies. Second, it brought together the use of hierarchical polynomial regression to examine the magnitude of the association between youth and parent ratings of aggression in relation to attachment anxiety and avoidance, as well as the use of difference scores to explore youth over- versus under-estimation of aggression relative to parent reports. As Berger et al. [13] note, multiple indices are useful in elucidating the nature and scope of attachment related biases. As previously discussed, a stronger or weaker association between youth and parent ratings of aggression does not necessarily preclude youth biases toward under- or overestimation of aggression relative to parent reports. Using linear regressions, we found that youth attachment anxiety was associated with youth reporting higher levels of parent-directed aggression than was reported by their parents. As previously noted, anxious attachment reflects hyperactivation of the attachment system, escalated expression of attachment needs, and an approach-based orientation in relationships. Our results suggest that youth with high attachment anxiety are likely sensitive about their aggression toward their parents, which would undoubtedly trigger even more anxiety [4], leading to higher reports of these behaviors compared to parents. Yet, youth anxiety was not related to youth reporting higher levels of parent-perpetrated aggression than that reported by parents.

In contrast, while youth attachment avoidance was not related to youth reporting higher or lower levels of aggression toward parents (compared to parent reports), avoidance was related to youth reporting higher levels of parent-perpetrated aggression (compared to parent reports). Intriguingly, as previously discussed, attachment avoidance is related to deactivation of the attachment system. Our results are interesting in that they suggest that even though the expression of attachment needs might be muted in youth high in attachment avoidance, they are no less sensitive to and may overestimate parental aggression toward them. Indeed, their sensitivity to detecting such aggression is likely key in perpetuating avoidance and masked expressions of attachment needs. In turn, their parents may lack awareness of the impact of their aggressive behavior on their adolescent and, consequently, fail to alter their behavior [45].

Taken together, these two indices suggest that youth attachment insecurity may introduce both common and unique biases in parent-youth perceptions of aggression. Specifically, attachment anxiety was related to a stronger association between youth and parent reports of youth-to-parent aggression. Furthermore, both youth attachment anxiety and avoidance were related to overestimating youth-to-parent and parent-to-youth aggression, respectively, relative to parents. Importantly, these two sets of findings are congruent with attachment theory and the conceptualization of attachment anxiety versus attachment avoidance.

### Clinical Implications

Understanding that parents and youth differ in their reports of parent and youth behaviors is critical to assessing and responding to the clinical needs of families [46]. The source of these discrepancies can range from contextual influences (e.g., ratings based on home or school observation), to the types of information being assessed, and critical affective, cognitive, or interpersonal factors at play. Traditionally, we have viewed divergent reports as a nuisance, clouding our ability to discern an objective truth, and in some instances this is true. Yet recent work has pointed to richness of discrepancies in elucidating the underlying challenges in communication experienced in dyadic or family relationships and the need for tailored clinical approaches [42].

Youth attachment anxiety may present different challenges in youth and/or family therapy than youth attachment avoidance. While youth with attachment anxiety tend to overestimate their aggression toward their parents, our results suggest that the association between youth and parents reports of youth aggression grows stronger as attachment anxiety increases. This shared perspective offers a starting point for exploring the attachment-related meanings of youth aggression in their relationship with parents, and alternative ways that youth might learn to cue their parents about their needs, as well as the importance of sensitive parent responsiveness. Our results regarding youth attachment avoidance were less clear and failed to reach significance, however the directions of our findings were consistent with the view that as youth avoidance increases, youth and parent reports of aggressive behavior increasingly diverge. High attachment avoidance was also related to youth overestimating their parents’ aggression toward them, relative to their parents’ reports. At its core, attachment avoidance represents the tendency to deactivate the attachment system and to distance oneself from others to avoid anticipated rejection or more. As a result, development of a therapeutic relationship with a youth, or within family therapy can be very challenging and must first focus on establishing shared trust and a shared understanding within family communication.

### Limitations and Future Directions

Although this study offers some valuable insight, it is not without limitations. First, our study focused on how youth attachment was associated with informant discrepancies, but did not speak to how parent attachment influences their reporting of intrafamilial violence. Attachment is inherently a relational construct and is best understood through research that adopts a dyadic and transactional framework to understand how interpersonal interactions shape communication and attachment relationships over time. Further research is needed to better understand these processes. Second, the present sample was comprised mostly of mothers (86%), which limits generalizability to father–child relationships. Third, this study examined the role of youth attachment anxiety and avoidance in relation to youth and parent reports of intrafamilial aggression in a sample of families where youth have clinically significant levels of mental health problems. While attachment related biases should be evident in samples where youth exhibit fewer behavioral problems, the magnitude of these effects might be less pronounced because of lower levels of attachment insecurity, intrafamilial aggression, or both. Replications with community samples of families would be needed to evaluate whether these findings generalize beyond families of youth with clinical levels of behavioral problems. Conversely, parents included in our sample had enrolled in a parenting group (but were yet to start the intervention) and may have been more attuned to their children’s emotional or behavioral than parents in comparable situations who did not seek this service. Lastly, the results of our study are correlational, so we cannot rule out the possibility that the informant discrepancies we detected were not caused by factors other than youth–parent attachment insecurity, such as family functioning.

## Conclusion

In summary, this study provided new insights into how youth attachment insecurity is associated with discrepancies in youth versus parent reports of intrafamilial aggression using a clinical sample. These findings demonstrate that some of these discrepancies are systematically related to youth attachment insecurity. We found that high levels of youth attachment anxiety were associated with fewer absolute discrepancies between parent and youth reports of youth-to-parent aggression. We also found that youth attachment anxiety was associated with youth over-reporting youth-to-parent aggression (relative to parents), whereas attachment avoidance was associated with youth over-reporting parent-to-youth aggression (relative to parents). Our findings suggest that service providers ought to consider adolescents’ attachment with their caregivers to contextualize potentially systematic discrepancies between youth and other informants when assessing and evaluating the progress of their youth clients.

## Summary

A growing body of research on youth social-emotional development has highlighted the importance of understanding the systematic sources of discrepancies between informants, especially parents and youth. In this study, we investigated whether youth attachment anxiety and avoidance were associated with discrepancies between parents and youth with clinically significant levels of mental health problems (*N* = 510 dyads; youth *M*_age_ = 13.96 years, range = 7.34–19.04). Unlike previous studies, parent and youth dyads reported on aggression they overtly perpetrated towards each other, meaning that discrepancies could not be attributed to informants being (un)able to observe those they are reporting on in different domains (e.g., parents being unable to observe youth aggression at school as well as at home).

Both parents and youth reported on parent-to-youth and youth-to-parent physical and psychological aggression via the Revised Conflict Tactics Scale (CTS-2), and youth reported on their attachment anxiety and avoidance with their parent via the Adolescent Attachment Anxiety and Avoidance Inventory (AAAAI). We assessed informant discrepancies using two analytic techniques: polynomial regressions, which tested the absolute magnitude of association between youth and parents, as well as difference scores, which tested the direction of youth reports of the frequency of aggression relative to parent reports (i.e., did youth under- or over-report by comparison).

Using polynomial regressions, we found that dyads’ reports of youth-to-parent aggression showed more agreement at high versus low levels of attachment anxiety. Using difference scores, we also found that youth attachment anxiety was associated with youth over-reporting (relative to parents) on youth-to-parent and parent-to-youth aggression, whereas attachment avoidance was associated with youth over-reporting (relative to parents) on parent-to-youth aggression. The findings point to the importance of understanding attachment as a source of informant discrepancies between in social-emotional development and family functioning.

## Data Availability

The data and materials that support the findings of this study are available upon reasonable request from the last author.
